# Identifying drivers of breast cancer metastasis in progressively invasive subpopulations of zebrafish-xenografted MDA-MB-231

**DOI:** 10.1186/s43556-022-00080-5

**Published:** 2022-05-26

**Authors:** Jerry Xiao, Joseph R. McGill, Apsra Nasir, Alexander Lekan, Bailey Johnson, Devan J. Wilkins, Gray W. Pearson, Kandice Tanner, Hani Goodarzi, Eric Glasgow, Richard Schlegel, Seema Agarwal

**Affiliations:** 1 Department of Pathology, Center for Cell Reprogramming, Georgetown University, Washington, DC USA; 2 Lombardi Comprehensive Cancer Center, Georgetown University, Washington, DC USA; 3 Hemostasis Branch, Division of Plasma Protein Therapeutics, Office of Tissues and Advanced Therapies, Center for Biologics Evaluation and Research, US Food and Drug Administration, Silver Spring, MD USA; 4 Laboratory of Cell Biology, Center for Cancer Research, National Cancer Institute, National Institutes of Health, Bethesda, MD USA; 5 Eastern Virginia Medical School, Norfolk, VA USA; 6 Department of Biochemistry and Biophysics, University of California, San Francisco, CA USA

**Keywords:** Zebrafish, Breast cancer, Zebrafish xenograft, Metastasis, Invasion

## Abstract

Cancer metastasis is the primary cause of the high mortality rate among human cancers. Efforts to identify therapeutic agents targeting cancer metastasis frequently fail to demonstrate efficacy in clinical trials despite strong preclinical evidence. Until recently, most preclinical studies used mouse models to evaluate anti-metastatic agents. Mouse models are time-consuming and expensive. In addition, an important drawback is that mouse models inadequately model the early stages of metastasis which plausibly leads to the poor correlation with clinical outcomes.

Here, we report an in vivo model based on xenografted zebrafish embryos where we select for progressively invasive subpopulations of MDA-MB-231 breast cancer cells. A subpopulation analogous to circulating tumor cells found in human cancers was selected by injection of MDA-MB-231 cells into the yolk sacs of 2 days post-fertilized zebrafish embryos and selecting cells that migrated to the tail. The selected subpopulation derived from MDA-MB-231 cells were increasingly invasive in zebrafish. Isolation of these subpopulations and propagation in vitro revealed morphological changes consistent with activation of an epithelial-mesenchymal transition program. Differential gene analysis and knockdown of genes identified gene-candidates (DDIT4, MT1X, CTSD, and SERPINE1) as potential targets for anti-metastasis therapeutics. Furthermore, RNA-splicing analysis reinforced the importance of BIRC5 splice variants in breast cancer metastasis. This is the first report using zebrafish to isolate and expand progressively invasive populations of human cancer cells. The model has potential applications in understanding the metastatic process, identification and/or development of therapeutics that specifically target metastatic cells and formulating personalized treatment strategies for individual cancer patients.

**Supplementary Information:**

The online version contains supplementary material available at 10.1186/s43556-022-00080-5.

## Introduction

Approximately 90% of all cancer-related deaths are attributed to metastasis [1]. Cancer metastasis is frequently associated with treatment failure, poorer prognosis, and high mortality [2]. In breast cancers, the presence of metastatic nodules in patients reduces 5-year expected survival rates to a dismal 28%, compared to 99% survival in localized breast cancer [2]. Unfortunately, despite nearly 150 years of research and repeated attempts to identify a metastasis-preventing therapy, candidate drugs that appear promising in preclinical studies repeatedly fail in the clinic [3, 4]. These clinical failures are likely due to a lack of accurate preclinical models for the study of metastasis [3, 4].

The first in vivo model for metastatic disease was developed in the 1970s by Fidler and Kripke, who injected B16 melanoma cells into immunocompromised mice [5]. These models are largely based on the preferential seeding of distant metastases through direct injection of xenografted cells into the circulatory system [6]. The metastatic cascade involves five stages: (1) invasion of the basement membrane and cell migration; (2) intravasation into the vasculature or lymphatic system; (3) survival in circulation; (4) extravasation from vasculature to secondary tissues; and (5) colonization of the secondary tumor sites [1]. Direct injection of cells into the mouse circulatory system forgoes stages 1–2 of the metastatic cascade, risking an overestimation of the population of cells that would successfully complete metastasis [6]. Furthermore, all rodent-based models require many cells for injection (~ 10^5^–10^6^ cells) and have long latency periods (~ 6–12 months) before tumor formation [6, 7]. The latter makes the rodent models unsuitable for personalized medicine applications (e.g., selecting treatment regiments tailored to an individual patient) [6].

In recent years, zebrafish (*Danio Rerio)* have emerged as a viable in vivo alternative to rodent models for studying cancer metastasis [8, 9]. Furthermore, zebrafish and humans share an obvious gene ortholog in 70% of all human genes, including 82% of all disease-related genes annotated in the Online Mendelian Inheritance in Man (OMIM) database [10]. Zebrafish models xenografted with human melanoma, prostate, salivary, and breast cancers have all developed tumors with histopathological similarities to their in-human counterparts [11–14]. We and others have also shown that xenografted cancers in zebrafish display similar chemotherapy sensitivity and therapeutic responses when compared to rodent models [13, 15–17]. In addition to fulfilling the criteria for being a suitable in vivo model system, zebrafish also offer the following advantages over rodent models: (1) Although zebrafish have a robust innate immune system in the first 2 weeks, they have not yet developed a mature adaptive immune system, therefore requiring minimal genetic manipulation for successful engraftment of human cancer cells [18]. (2) Cells engrafted in 2 days post-fertilized (dpf) zebrafish can survive for up to 8 days [19]. (3) Zebrafish require the transplantation of a few hundred cells as opposed to millions of cells needed for a single mouse [8, 20]. (4) Transgenic zebrafish lines have been engineered to express fluorescent vasculature allowing for non-invasive, real-time imaging of cancer cell invasion and circulation [21, 22]. (5) The cost of zebrafish husbandry in a vivarium is significantly less than those required for rodents [23]. (6) Each zebrafish breeding pair can yield as many as 300 embryos allowing for rapid and robust scalability of experiments [16, 24]. Given these advantages, zebrafish xenografts are primed to become a critically important tool within a cancer researcher’s arsenal.

In this study, we used xenografted zebrafish embryos to identify two subpopulations of MDA-MB-231 breast cancer cells that exhibited progressively invasive behavior. These populations are analogous to circulating tumor cells found in human cancers [25, 26]. Differential gene analysis of these subpopulations with their parental population revealed activation of an epithelial-mesenchymal transition (EMT) program as well as enrichment of several pathways consistent with prior reports of breast cancer metastasis. In addition, differential RNA-splicing analysis also linked splicing events in BIRC5 and other genes to an invasive phenotype. Finally, we demonstrate that knockdown of the genes DDIT4, MT1X, CTSD, and SERPINE1 is sufficient to reduce MDA-MB-231 cellular invasion, suggesting an opportunity for drug development targeting these genes. Taken altogether, this study is leverages xenografted zebrafish to identify candidate drivers of breast cancer cellular invasion.

## Results

### Optimization of zebrafish xenografts for the selection of invasive subpopulations

**Fig. 1:**
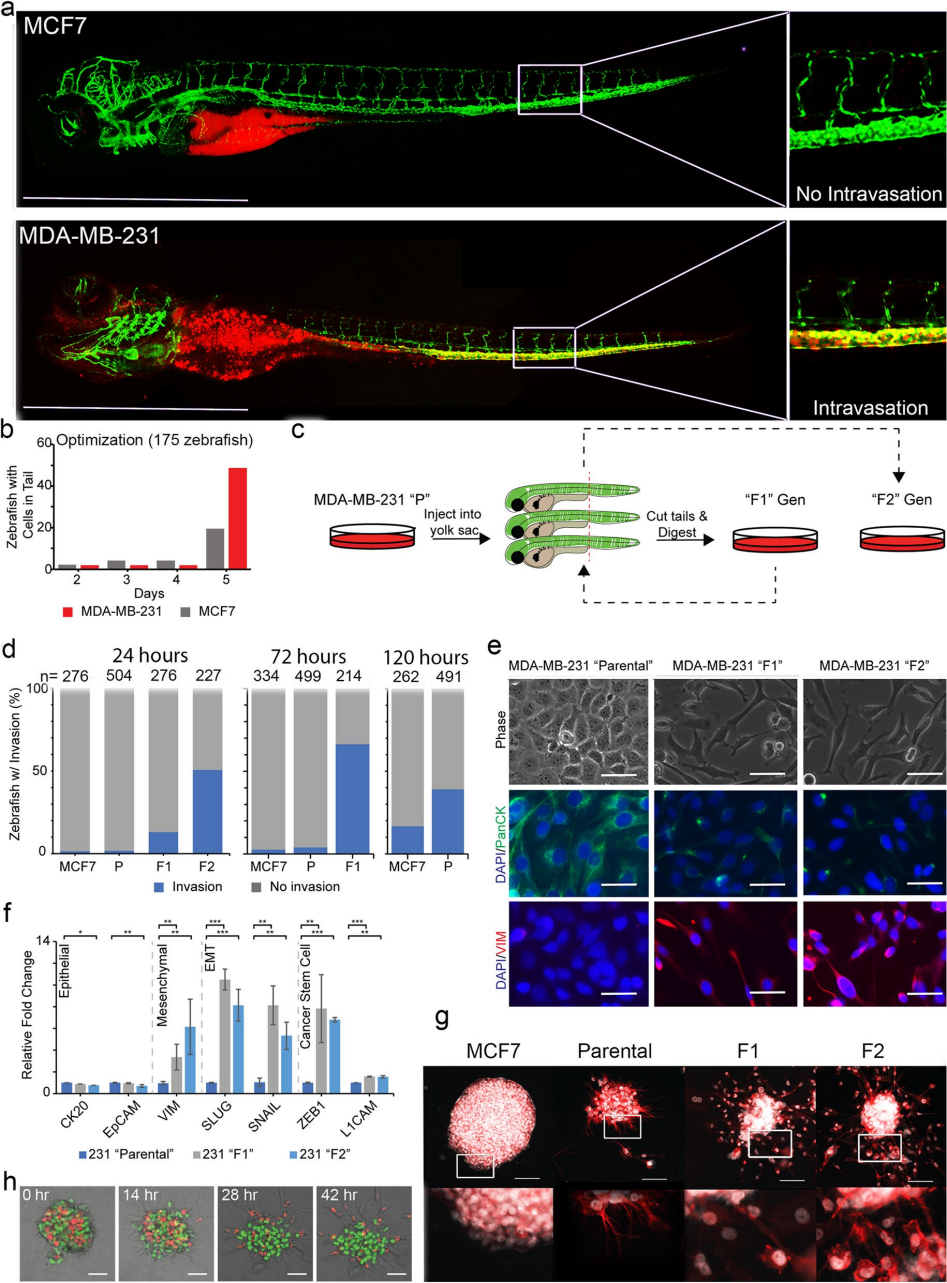
Labeled MDA-MB-231 cells were injected into the yolk sac of 2-days post fertilized zebrafish embryos and monitored for invasion. **a** Injection of MCF7 and MDA-MB-231 cells into the yolk sac resulted in cell arrest within the caudal plexus. Images of zebrafish were taken at 4x magnification using Olympus IX-71 inverted fluorescence microscope. **b** Transiently labeled cells arrest within the tail of the zebrafish within 5 days of injection. **c** Workflow of serial transplantation of the MDA-MB-231 heterogeneous parental population to generate the F1 and F2 subpopulations. **d** MDA-MB-231 F1, and F2 cells arrest within the tails of zebrafish progressively faster. Three separate experiments evaluating the parental (“P”), F1, and F2 ability to invade over three time points (120, 72, and 24 hours). Xenografted parental, F1, and F2 cells appeared in ≥40% of injected zebrafish in 120, 72, and 24 hours, respectively. **e** Phase and immunofluorescence images of resulting in vitro cultured MDA-MB-231 parental, F1, and F2 cells. **f** qRT-PCR amplification of epithelial (CK20, EpCAM), mesenchymal (VIM), EMT TFs (SLUG, SNAIL), and cancer stem cell markers (ZEB1, L1CAM) was performed of the three subpopulations. Relative fold changes were normalized to MDA-MB-231 parental cells. * = *p*-value < .05; ** = *p*-value < .001; *** = *p*-value < .0001 (**g**) Clusters of MCF7, MDA-MB-231 parental, F1, and F2 cells were embedded within a 3-dimensional Matrigel-based extracellular matrix and allowed to invade over 24 hours. Clusters were stained for nuclei (white) and phalloidin (red). **h** RFP-labeled F2 and GFP-labeled parental cells were co-clustered and embedded in 3D ECM showing F2 cells invade the ECM progressively with time, but not parental cells. Scale bars = 100 μm

We first sought to establish that cell transplantation into 2dpf zebrafish could differentiate between cells that are expected to intravasate and those that are not. We injected cells from two human epithelial breast cancer cell lines, MCF7 and MDA-MB-231, into the yolk sac of 2dpf zebrafish embryos. In mouse models, the breast adenocarcinoma MCF7 cell line has consistently been shown to be poorly metastatic [27]. On the other hand, MDA-MB-231 cells, which were derived from the pleural effusion of a patient with invasive triple-negative ductal carcinoma, are routinely used as a cellular model for aggressive late-stage cancer [28, 29]. Based on our prior studies, each zebrafish yolk sac could be injected with up to 200 cells to allow for both high-throughput screenings while also maintaining zebrafish viability [13, 30]. Transiently labeled MCF7 or MDA-MB-231 cells were observed within the tail of zebrafish (2/75 MCF7-xenografted, 2/147 MDA-MB-231 xenografted fish) within 24 hours. By day 5, labeled xenografted MCF7 and MDA-MB-231 cells were observed in the tails of 16/75 (21.33%) and 59/147 (40.14%) zebrafish, respectively (Fig. 1a, b).

### Selection of invasive MDA-MB-231 subpopulations

Based on these initial results, we designed a study to select for the subpopulation of MDA-MB-231 cells that have invasive potential (Fig. 1c). *flk:gfp* zebrafish are a transgenic zebrafish line carrying a germline-integrated GFP-transgene designed to outline the zebrafish vasculature [31]. We injected MDA-MB-231 cells into the yolk sacs of 100 transgenic *flk:gfp* labeled zebrafish. Cells that migrated to the tail within 5 days (F1 generation) were isolated, pooled, expanded and re-injected into an additional 100 zebrafish and cells that migrated to the tails were again isolated and expanded in vitro (F2 generation). Finally, pooled cells from the F2 population were injected into the yolk sacs of 2dpf zebrafish to evaluate their invasive phenotype. While < 40% of zebrafish injected with parental cells exhibited intravasation after 120 hours, > 50% of F1- and F2-injected zebrafish showed intravasation into the tail within 72 and 24 hours, respectively (Fig. 1d). Short tandem repeat (STR) analysis confirmed that both F1 and F2 cell populations were derived from the same parental MDA-MB-231 lineage and is similar to ATCC database for STR of this cell line (Supplementary Table 1).

Next, we compared the morphologies of cell cultures generated from parental, F1, and F2 generations (Fig. 1e). While the parental cultures exhibited a cobblestone appearance commonly associated with epithelial cells, F1 and F2 cultures revealed a protruded, spindle-like morphology associated with invasive mesenchymal cells [32]. This observed epithelial-to-mesenchymal (EMT) phenotype change was also confirmed via immunofluorescence. F1 and F2 cells showed increased expression of the mesenchymal marker and decreased expression of the epithelial marker relative to parental cells (Fig. 1e). Furthermore, quantitative reverse-transcriptase PCR (qRT-PCR) revealed that F1 and F2 cells exhibited a statistically significant decrease in expression of epithelial markers (CK20, EpCAM), and increased expression of mesenchymal (VIM), EMT transcription factors (SLUG, SNAI1) and cancer stem cell markers (ZEB1, L1CAM) (Fig. 1f). Cells maintained and constantly expanded in standard culture conditions over 2 years retained these differences, suggesting the stable nature of changes within these three populations.

We further sought to evaluate whether the progressively invasive behavior of the F1 and F2 generations were reproducible in an in vitro invasion assay. MCF7 cells, parental cells, and the F1 and F2 generations of MDA-MB-231 cells were clustered in suspension culture and embedded within a 3-dimensional extracellular matrix composed of Matrigel and collagen I [33, 34]. Invasion in the extracellular matrix was monitored via immunofluorescence staining of phalloidin and the cell nuclear bodies. As expected, MCF7 clusters did not exhibit any invasion in this extracellular matrix after 24 hours (relative area of invasion = 0.00% ± 0.00%, *n* = 9 clusters). By contrast, the F1 clusters (relative area = 8.58% ± 9.77%, *n* = 15) and F2 clusters (relative area = 19.0% ± 13.0%, *n* = 23) of MDA-MB-231 cells exhibited 3- to 6-fold increases in invasion compared to the parental clusters (relative area = 3.87% ± 2.78%, *n* = 26) (Fig. 1 g). Furthermore, different modes of migration could be observed in these clusters. While parental clusters only form cytoplasmic protrusions from the cluster, clusters of F1 and F2 generations demonstrated clear migration of the entire cell body (Fig. 1 g). Finally, co-clustering of parental-GFP and F2-RFP labeled cells revealed that F2-RFP cells consistently localized to the edges of clusters, adopting a trailblazer cell phenotype in invasion (Fig. 1 h and Supplementary Video 1 )[33, 35].

### Selected F1/F2 populations of MDA-MB-231 cells are enriched for metastasis-associated genes and pathways

The repeated failures of metastasis-targeting therapeutics suggests that current models for studying metastasis may be inefficient at identifying the estimated .0001% of cells that will progress through any one stage of the metastatic cascade to the next [4]. Therefore, the ability to accurately identify this subpopulation of metastasis-prone cells could significantly improve the ability of drug screens to identify clinically useful metastasis-targeting therapeutics [4]. The F1 and F2 cells described here were selected such that they demonstrate an ability to intravasate and circulate. Therefore, transcriptomic differences between the parental, F1, and F2 populations of MDA-MB-231 cells may allow the identification of pathways specifically involved in intravasation and circulation during breast cancer metastasis.

**Fig. 2:**
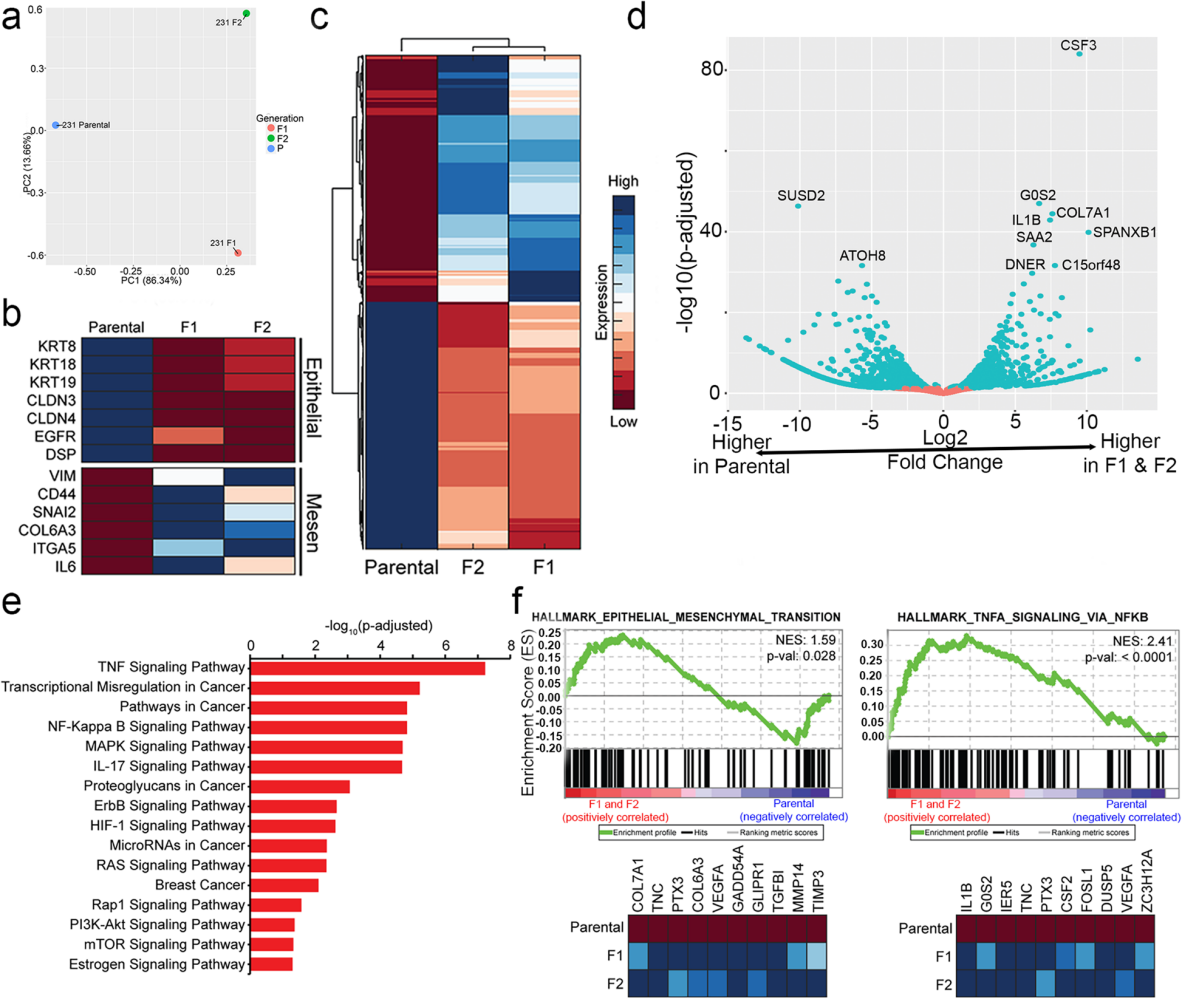
RNA-sequencing of MDA-MB-231 parental, F1, and F2 populations reveals increased expression of EMT genes and metastasis-associated genes in F1/F2 compared to parental cell populations. **a** Principal-component analysis reveals transcriptomic differences between all three subpopulations. **b** A heatmap depicting RNA-sequencing expression of a select panel of epithelial and mesenchymal markers is shown. **c** A clustergram of the overall transcriptomic landscape of the parental, F1, and F2 populations. **d** A volcano plot depicting the most significantly upregulated and downregulated genes when comparing the F1 and F2 populations relative to the common parental control. **e** A KEGG analysis was performed, revealing enrichment of several important cancer-associated pathways in the F1 and F2 populations. **f** GSEA analysis identified enrichment of the hallmarks gene set Epithelial-Mesenchymal Transition and TNFα Signaling via NF-kβ, among other gene sets

Principal component analysis of sequenced RNA libraries from the three populations (parental, F1, and F2) of MDA-MB-231 cells revealed significant transcriptomic changes between all three populations (Fig. 2a). A select panel of EMT-associated genes was also identified based on a literature review [36, 37]. Consistent with the observed phenotypes in culture, the F1 and F2 populations of MDA-MB-231 cells expressed lower levels of epithelial (KRT8, KRT18, KRT19, CLDN3, CLDN4, EGFR, and DSP) and higher levels of mesenchymal (VIM, CD44, SNAI2, COL6A3, ITGA5, IL6) genes compared to the parental cells, indicating activation of an EMT program in the F1 and F2 populations (Fig. 2b).

Genes were identified as significantly differentially expressed in the F1 and F2 populations relative to parental cells based on a criterion of: (1) an absolute log 2-fold change cutoff of at least 1.5, and (2) an adjusted *p*-value cutoff of 0.05. In total, 842 genes were found to be upregulated and 875 genes were downregulated in the F1 and F2 populations (Fig. 2c). Among the genes that were most significantly upregulated were some that have been previously identified as drivers of breast cancer metastasis, e.g. CSF 3[38], G0S 2[39], COL7A 1[40], IL1 6[41], and DNER [42]. In addition, we identified differential expression of genes that have previously not been reported to be associated with metastasis in breast cancers. For instance, SPANXB1 had previously been implicated as an immunogenic tumor antigen in breast cancer [43] while SAA 2[44] and C15orf4 8[45] were associated with poorer prognosis in breast cancers. However, none of these genes have been implicated as drivers of breast cancer metastasis.

Pathway enrichment analysis can be used to provide potential mechanistic insights using our experimental data [46]. The Kyoto encyclopedia of genes and genomes (KEGG) is a publicly available database of curated gene sets containing various cancer-associated gene sets as well as common signaling pathways [47]. Using the parental population as a common control, KEGG analysis identified enrichment of several cancer-associated gene sets in the F1 and F2 populations relative to the parental cells. The cancer-associated gene sets identified in the F1 and F2 generations of MDA-MB-231 cells included transcriptional misregulation in cancer (hsa05202), pathways in cancer (hsa05200), and breast cancer (hsa05224) (Fig. 2e). In addition to these cancer-specific gene sets, the F1 and F2 populations were also significantly enriched for TNFα [48], MAPK [49, 50], IL-1 7[51], ErbB [52], HIF- 1[53], RAS [54], RAP 1[55], PI3K-AKT [56], and mTOR [56] signaling pathways, all of which have previously associated with breast cancer metastasis (Fig. 2e).

Gene Set Enrichment Analysis (GSEA) is another computational method commonly used to determine whether there is a significant difference in pathway expression between biological states [57, 58]. Once again using the parental cell population as a common control, GSEA analysis identified enrichment of several gene sets in the F1 and F2 populations relative to their parental source, including the hallmark gene set EMT (NES = 1.59, *p*-value = 0.028) and TNFα signaling (NES = 2.41, *p*-value < .0001) (Fig. 2f and Supplementary Table 3). These results are in agreement with those obtained from KEGG analysis.

Taken together, differential gene analysis and pathway enrichment comparing the F1/F2 populations against their parental population were consistent with previously reported findings [48–56], providing sufficient confidence that the zebrafish xenograft model used here could accurately and adequately identify genes, pathways and genesets important for cellular invasion and metastasis.

### Differential RNA-splicing in BIRC5 may be involved in breast cancer invasion

**Fig. 3:**
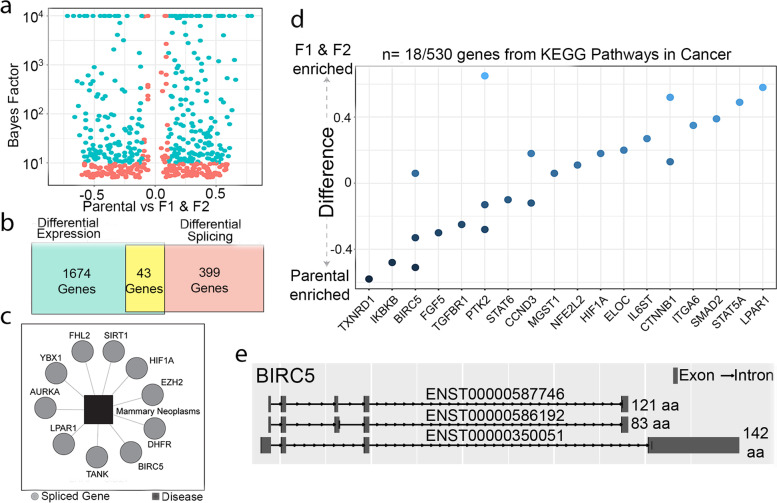
Differential RNA-splicing and protein-protein interactions associated with cancer and metastasis were enriched in the F1 and F2 populations compared to parental population. **a** A volcano plot of the 526 differentially spliced transcripts. **b** Overall, 43 genes were both significantly differentially expressed and differentially spliced. **c** Gene-disease association analysis using DisGeNET revealed differentially spliced genes associated with mammary neoplasms. **d** KEGG pathway analysis identified differentially spliced genes included within the Pathways in Cancer geneset. **e** BIRC5 was one of the few genes that was both differentially expressed and differentially spliced. The three spliced variants of BIRC5 differentially spliced in the F1 and F2 populations are shown

Given the limited absolute number of genes encoded by the human genome, differential RNA-splicing, which can result in variations of proteins given a similar genomic template, might play a critical role in breast cancer metastasis [59]. We identified a total of 526 differentially spliced products spanning 442 unique genes when comparing the transcriptomes of the F1/F2 and parental generations of MDA-MB-231 cells (Fig. 3a and Supplementary Table 4). However, only 43 genes displayed both differential expression and differential splicing (Fig. 3b). Among the 442 genes with splice variants, DisGeNET, an integrated platform for evaluating gene-disease associations [60], identified several genes with strong evidence of involvement in mammary neoplasms, including HIF1A, BIRC5, LPAR1, SiRT1 (Fig. 3c). A KEGG pathway analysis of the 442 genes also identified 18 additional genes with differential splice variants that are also included in the KEGG pathways in cancer (hsa05200) gene set.

Of the genes identified, BIRC5 stood out as a candidate gene as it was identified in both the DisGeNET and KEGG analyses. BIRC5 splice variants have been implicated in affecting a wide range of cellular functions. For example, variant ΔEx3 has been identified as an anti-apoptotic protein, while variant 2b, which has a truncated BIR domain, has pro-apoptotic effects [61]. BIRC5 splicing is frequently identified in breast cancers and has been suggested as a potential chemotherapeutic target [62]. In this work, we identified three variants: (1) survivin-2b + 32 (diff = 0.06), (2) survivin-ΔEx3 (diff = − 0.33), and (3) an unnamed, 105 amino-acid variant that retains intron 3 (diff = − 0.51 )[61, 63] (Fig. 3e). Other genes with multiple splice variants that were differentially expressed in the F1 and F2 generations of MDA-MB-231 cells include AP1G1, CHD8, COPB2, IMPDH1, IRAK1, LIPA, MICAL2, NR1H3, PTK2, SEPTIN2, SERPINA1, SLC41A3, AND TNIP1 (Fig. 3e).

### Zebrafish assays identify functionally significant drivers of cellular invasion

Next, we sought to determine whether the increased invasiveness of the F1 and F2 populations could be inhibited through an in vitro functional study. In the functional study, we included genes that (a) showed significantly increased expression in the F1 and F2 populations relative to the parental population and (b) spanned a variety of cellular functions. Based on these criteria, we identified seven genes to evaluate in an in vitro functional assay: (1) CTSD, a proteolytic activator [64]; (2) DDIT4, an mTORC1 inhibitor [65]; (3) MT1X, a metallothionein [66]; (4) S100A11, a cell cycle regulator [67, 68]; (5) SERPINE1, a fibrinolysis inhibitor [69]; (6) SNRPA1, an epigenetic modulator in breast cancers [59], and (7) SRGN, a mediator for cellular apoptosis [70].

**Fig. 4:**
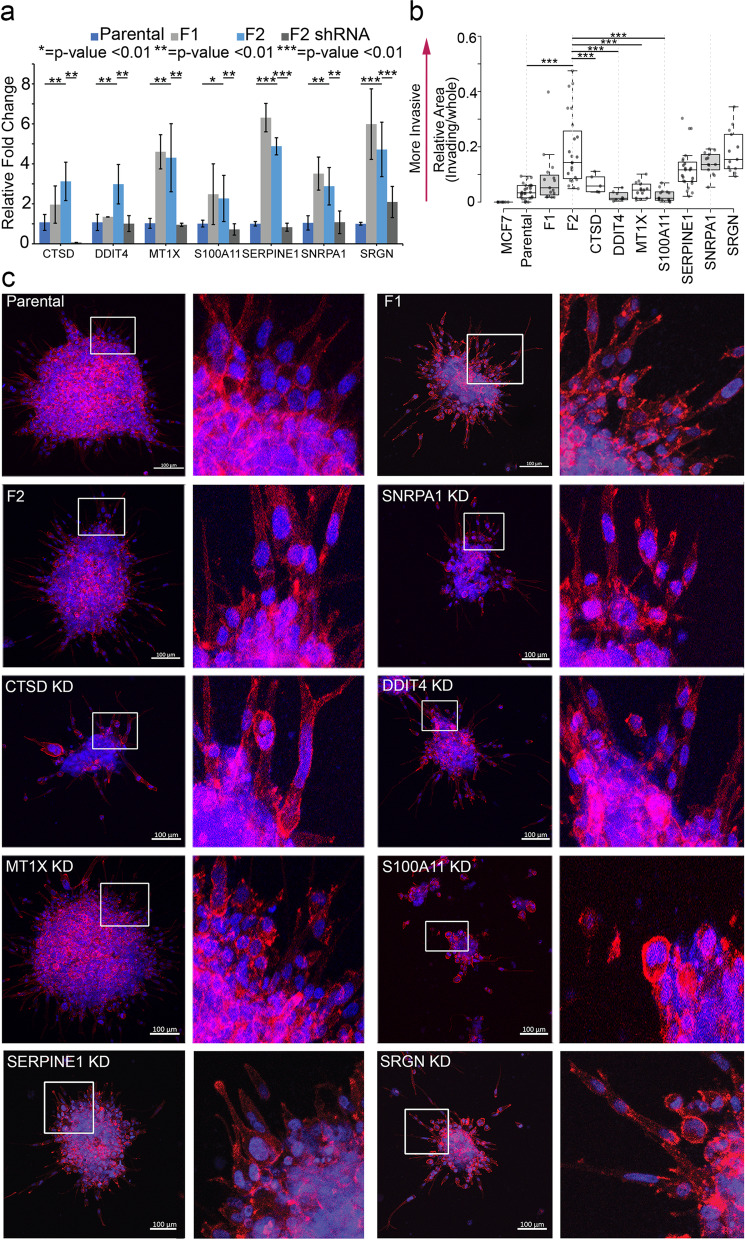
Knock-down of DDIT4, MT1X, SERPINE1 and CTSD in MDA-MB-231 F2 cells revealed a role in breast cancer invasion. **a** qRT-PCR amplification quantifying knockdown of the respective gene within the MDA-MB-231 F2 population. Notably, expression of all genes was reduced to near parental expression levels. **b** After 24 hours, ECM embedded cell clusters of various knockdown and wildtype lines were quantified using ImageJ for invasion. Invasion is measured as relative area of cells that escape the cluster. **c** Representative immunofluorescence images (left panel of each pair) of embedded clusters stained for Hoechst (blue) and Phalloidin (red). Digitally zoomed insets taken from the full-sized image and highlighting invasion at the borders are provided (right panel of each pair). Insets correspond to dashed boxes on original scale image. Scale bars = 100 μm. * = *p*-value < .01; ** = *p*-value < .001; *** = *p*-value < .0001

We sought to determine whether knockdown of the target genes in the F2 generation of MDA-MB-231 cells by short hairpin RNA (shRNA) could reduce cellular invasion to levels comparable to those observed in the parental MDA-MB-231 population. Following shRNA-mediated knockdown, we demonstrated using qRT-PCR significantly decreased expression of the target genes in the F2 cell lines (Fig. 4a). Specifically, shRNA targeting of CTSD resulted in a 46-fold decrease in gene expression compared to untargeted F2 cells (Fig. 4a). Less dramatic but significant decreases in gene expression were observed following knockdown of DDIT4 (2.94-fold decrease), MT1X (4.52-fold decrease), S100A11 (3.14-fold decrease), SERPINE1 (5.88-fold decrease), SNRPA1 (2.67-fold decrease), and SRGN (2.25-fold decrease) (Fig. 4a).

Following gene knockdown, we embedded clusters of each cell line into a 3-dimensional ECM analog in vitro and evaluated their invasion via Hoescht and phalloidin staining after 24-hours (Fig. 4b, c). As controls, we show that both parental (3.8 ± 2.77% relative area of invasion) and MCF7 (0 ± 0%) clusters demonstrated significantly (*p*-value < .0001) lower invasion relative to the F2 clusters (19.0 ± 13% relative area of invasion) (Fig. 4b). Knockdown of the following genes also resulted in significantly (*p*-value < .0001) reduced relative areas of invasion to the F2 generation of MDA-MB-231 cells: (1) CTSD (6.6 ± 3.3%), (2) DDIT4 (2.1 ± 1.7%), (3) MT1X (4.3 ± 2.7%), (4) S100A11 (2.3 ± 2.1%) and (5) SERPINE1 (12 ± 7.4%).

Finally, we sought to determine whether the genes we targeted in the in vitro invasion assay were clinically relevant in metastatic breast cancer. Using publicly available clinical and gene expression data from the METABRIC study [71] consisting of 1904 breast cancer patients, the computational tool X-tile was used to determine an appropriate cut-off z-score value for determining high- or low-expression of the genes of interest [72]. Kaplan-Meier survival and Cox proportional hazards analyses were performed to evaluate associations between high expression of the respective genes of interest and overall patient survival (Supplementary Fig. 1). Of the seven genes evaluated, increased expression of the following genes showed statistically significant (*p*-values < .05) associations with shorter overall survival (expressed as a hazard ratio (HR) over 240 months): (1) CTSD (HR = 1.3), (2) DDIT4 (HR = 1.1), (3) MT1X (HR = 1.6), (4) SERPINE1 (HR = 1.9), and (5) SRGN (HR = 1.8) (Supplementary Fig. 1). Though not statistically significant, higher expression of either S100A11 (HR = 0.88, *p*-value = 0.08) or SNRPA1 (HR = 0.86, *p*-value = 0.093) were associated with longer survival, suggesting a more complex relationship between these genes and metastasis (Supplementary Fig. 1). Therefore, these data suggest that CTSD, DDIT4, MT1X, and SERPINE1 represent a set of relevant targets that could be further evaluated as biomarkers and/or gene targets to for breast cancer anti-metastasis therapeutics.

## Discussion

We report the use of zebrafish xenografts to generate three subpopulations of MDA-MB-231 cells with progressively increased invasiveness. We have successfully isolated and enriched the subpopulation of MDA-MB-231 cells that migrate to the tail of 2dpf zebrafish embryos following injection into yolk sacs (Fig. 1). The cells obtained from two cycles of enrichment and re-injection into zebrafish embryos (F1 and F2 generations) show a much more rapid migration (1- and 3-days for > 50% of zebrafish embryos injected, respectively) to the tail compared to the parental MDA-MB-231 cells (5-days for > 40% of zebrafish embryos injected). We consider these cells analogous to circulating tumor cells (CTCs). CTCs are a unique population of tumor cells that invade the basement membrane and intravasate into the vasculature, serving as the source of cells from which metastatic nodules will eventually form [26]. In this model, only those MDA-MB-231 cells that have the capacity to invade the basement membrane, intravasate into the zebrafish vasculature, and migrate using the circulatory system are monitored and studied. Furthermore, our finding that MCF7 cells (which lack metastatic potential [27]) do not exhibit similar invasiveness or migration to the tail, supports the use of this in vivo zebrafish model for studying metastatic cancers.

Additional evidence that the subpopulation of MDA-MB-231 cells that migrate to the zebrafish tail have characteristics of metastatic cancer cells was obtained using analyses of RNA-sequencing data (Fig. 2). Genetically, we quantified a divergence of gene expression between parental MDA-MB-231 cells and the cells that were enriched for intravasation and migration (F1 and F2 generation of MDA-MB-231 cells). Importantly, the F2 cells maintained in culture for over 2 years continued to show the same transcriptomic divergence. F2 cells maintained in culture also conserved the upregulation of genes associated with multiple signaling pathways (e.g. TNFα and PI3K-AKT signaling) associated with cancer cells (Fig. 2). Furthermore, genes associated with a cellular phenotype have limited value unless they are validated in a functional assay. Knockdown of selected genes in the invasive F2 cells was performed using shRNA (Fig. 4). We demonstrated that shRNA-mediated knockdown of CTSD, DDIT4, MT1X, and SERPINE1 genes within clusters of F2 cells resulted in a statistically significant reduction in the relative area of invasion compared to parental MDA-MB-231 cells. In previous mechanistic studies, CTSD has been shown to act to stimulate metastasis through various roles including promotion of mammary fibroblast outgrowth through LRP1 and inducing expression of ICAM- 1[64]. In gastric and prostate cancers, overexpression of DNA damage inducible transcript 4 (DDIT4) [65] and the metallothionein MT1X [66] have both been identified to associate with poorer prognosis and/or increased metastatic phenotypes. Among the four candidates, expression of the serine protease inhibitor SERPINE1 (AKA PAI-1) has been linked to paclitaxel resistance in TNBC [73] and as well as a key activator of the EMT program [74]. The overexpression of these four genes within the F2 population further suggests an important role for these genes in breast cancer metastasis.

Analysis of RNA-sequencing data also demonstrated that the F1 and F2 populations exhibited increased expression of genes that are known to be associated with cancer metastasis. For example, CSF3 (AKA granulocyte colony-stimulating factor, G-CSF) has been shown to act through multiple mechanisms to enhance breast cancer metastasis [38]. For instance, induction of granulocytic myeloid-derived suppressor cells which subsequently reduce T cell activation and proliferation or through the direct activation of H-Ras oncogene, MAPK, ERK1/2, and AKT signaling pathways [38]. Other genes found to be significantly upregulated in the F2 cells included a diverse geneset composed of G0/G1 Switch 2 (G0S2 )[39], COL7A 1[40], interleukin 16 (IL-16) [41], and delta/notch-like epidermal growth factor-related receptor (DNER) [42], all of which have been previously reported to enhance breast cancer metastases. For instance, previous studies have demonstrated that G0S2 activates the Hippo pathway and induces expression of various different matrix metalloproteinases (MMPs) [39]. On the other hand, elevated expression of neutrophil-derived IL-16 is frequently found within the premetastatic niche of lungs in murine mouse models studying breast cancer metastasis [41]. On the other hand, genes such as DNER affect breast cancer metastasis more directly through the activation of the EMT program [42].

In breast cancer, a subpopulation of “trailblazer” cells has been observed based on their ability to act as leader cells during collective invasion [33]. During invasion from a primary tumor, these trailblazer cells may induce invasion of non-trailblazer cells [33]. When co-clustered with parental MDA-MB-231 cells, F2 cells were consistently observed along the leading edge of invasion. This phenotype is consistent with the behavior of “trailblazer” cells [33]. Furthermore, many of the genes showing upregulation in the F2 cells have also been identified in breast cancer “trailblazer” cell populations, providing another line of evidence that the in vivo zebrafish model efficiently selects invasive cellular subpopulations [33].

In addition to the individual genes, we also identified several signaling pathways enriched in the F1 and F2 cells. Previous studies have identified these pathways in breast cancers and/or enhanced metastasis [75–78]. Some of these pathways, such as the PI3K/AKT, MAPK, and mTOR signaling pathways, are the targets of therapeutics approved for treatment of breast cancer patients (e.g. rapamycin )[79]. Notably, the most highly enriched pathway in the F1 and F2 cells was TNFα signaling (Fig. 2). TNFα is an essential pro-inflammatory cytokine commonly found in the tumor microenvironment and frequently involved in a pro-metastatic role in breast cancers [80]. Others have previously reported that inhibition of TNFα via therapeutic monoclonal antibody Infliximab reduces MDA-MB-231 invasiveness by 41–60 %[81]. Unfortunately, therapeutic inhibition of TNFα in breast cancers have been particularly controversial, with several studies purporting concerns of increased breast cancer progression following TNFα treatment and competing studies indicating a lack of association with breast cancer recurrence [82]. Controversial scientific issues such as these could benefit greatly from the rapid experimental turnaround times and scalability of the zebrafish model and workflow described here. Quantitative results obtained using a large number of zebrafish would provide valuable preclinical evaluation of the safety and efficacy of TNFα inhibitors in the treatment of metastatic breast cancers.

Recently, attention has also turned towards the role that alternative splicing—the process by which multiple functionally distinct transcripts can be encoded from a single gene—plays in breast cancer metastasis [83]. In-depth studies have identified global alternative splicing signatures associated with epithelial-mesenchymal transition [84] and breast cancer metastases [59]. In this study, we identified differential alternative splicing in 526 transcripts spanning 442 unique genes. Of those genes with spliced variants were genes from the KEGG pathways in cancers gene set as well as those with strong associations to mammary neoplasms. For instance, the identification of BIRC5 splice variants in the F1/F2 populations is encouraging. BIRC5 variants have consistently been implicated in a broad range of cellular behaviors, including breast cancer invasion [62, 85]. Investigations of targeted therapeutic compounds against splice variants to inhibit cancer metastasis are already underway [61]. Validation studies that evaluate the effects of silencing specific BIRC5 splice variants in the F1 and F2 cells in zebrafish embryos could provide a model for a lower cost and rapid preclinical platform evaluating these compounds.

Finally, using zebrafish xenografts and the F2 population of MDA-MB-231 cells, we demonstrate that inhibition of CTSD, DDIT4, MT1X, or SERPINE1 singly can reduce cellular invasion in vitro (Fig. 4). Among these four candidates, CTSD is the most promising candidate for a metastasis-specific therapy. CTSD is a marker of poor prognosis in breast cancer [86]. Furthermore, studies have been carried out targeting CTSD with monoclonal antibodies in mice models using one MDA-MB-231 and two TNBC patient-derived xenografts. These studies demonstrated inhibition of tumor growth via natural killer cell activation and release of the anti-tumor cytokine IFNγ [86]. Moving forward, drug screens taking advantage of the high scalability and low cost of zebrafish assays could be performed to evaluate the anti-invasion efficacy of therapeutics targeting these four biomarkers.

In addition to evidence confirming that the F2 cells showed upregulation of genes and gene pathways associated with cancer or breast cancer metastases, we also report the novel finding that the SPANXB1 gene was upregulated in the F1/F2 subpopulations. The SPANXB1 gene is a member of the SPANX family of genes located in a cluster on chromosome X [43]. The SPANXB1 gene is known to play a role in spermatogenesis but has not previously been associated with cancer metastasis [43]. In triple negative breast cancers, SPANXB1 expression has been identified in circulating small extracellular vesicles and is thought to be acted upon by metastasis suppressor SH3GL2 [87]. Further comprehensive mechanistic studies are needed to determine the exact extent with which SPANXB1 is associated with cancer metastasis.

Despite the promising results reported here, there are some limitations to this pilot study. In cancer metastasis, not all cells that intravasate successfully will become metastatic nodes [88, 89]. In our current model, experiments were stopped 5 days after injection into zebrafish embryos to avoid complications associated with xenograft rejection [90]. A future workaround to extend the period of study is the use of genetically engineered adult immunodeficient zebrafish analogous to severe combined immune deficiency (SCID) mice which have been developed recently [91]. By extending the time frame of study using adult *prkdc*^*−/−*^*, il2rga*^*−/−*^ transgenic fish, we can permit the seeding and outgrowth of metastatic nodules as opposed to simple arrest within the tail. Despite lacking sites of secondary metastasis for breast cancer such as the brain, liver, and bone, adult zebrafish would allow the study of lung metastases, which is a major secondary site (~ 60% incidence) in breast cancer physiology [92]. Another variable that needs to be acknowledged involves differences in physiological temperature between zebrafish, which are bred and maintained at 26–28 °C, and humans. For this study, xenografts were maintained at 34 °C for the duration of their injection and monitoring, with no dramatic effects on zebrafish viability or xenograft behavior [15, 93].

Overall, this study provides a proof-of-concept that, once scaled to include additional cell lines and patient-derived samples [94], could significantly improve the ability to identify drivers of cancer metastasis. The experimental workflow reported here also offers a framework for evaluating genes (either singly or in combination) associated with metastases. Finally, our workflow provides a platform for the identification and preclinical evaluation of drugs that target metastases, a critically unmet need.

## Materials and methods

### Zebrafish husbandry, injections, and isolation

All animal procedures were conducted in accordance with NIH guidelines for the care and use of laboratory animals and approved all experimental protocols with zebrafish by the Georgetown University Institutional Animal Care and Use Committee, Protocol #2017–0078. For the evaluation of metastasis, cells were first labeled with the lipophilic dye CM-dil (Thermo Fisher, V22885) according to the manufacturer’s instructions. Zebrafish embryos were injected with 100–200 labeled tumor cells into the yolk sac at 2-day post fertilization (2dpf). *Tg (kdrl:grcfp)* zebrafish express green reef coral fluorescent protein in the vascular endothelium [21]. Injected embryos were arrayed in a 96 well plate and evaluated at 2–3 hour post injection to discard embryos from analysis if they showed any migration into the blood vessel as that would be indicative of problems with the injection process. Invasion into the vasculature was monitored on a day-to-day basis via an Olympus IX-71 inverted microscope. Embryos were evaluated daily for tumor cell migration and health of the embryos. On days of harvest, zebrafish was rinsed twice in autoclaved water followed by a brief exposure to ethanol to sterilize zebrafish. Zebrafish were then washed twice in primocin containing 1x PBS. Zebrafish were then incubated for 30 minutes in PBS mixed with 1x primocin, 1x plasmocin and Y-compound (1 μM). Tails and heads were cut using a scalpel and collected in cell culture medium supplemented with primocin and Y-compound (5 μM). Tails were rinsed three times in PBS supplemented with primocin and Y-compound and subsequently spun in a centrifuge at 300 g for 5 minutes at 4 °C and digested in a mixture of 500 μl of PBS and Liberase at 37 °C for 20 minutes. After 20 minutes, the digestion was stopped by adding 5 ml of complete DMEM and spun in a centrifuge at 300 g for 5 minutes at 4 °C and washed once more with PBS supplemented with primocin and Y-compound. Cells were subsequently plated in culture dishes with M-2D medium supplemented with primocin and plasmocin. Cell medium supplemented with primocin and plasmocin was changed daily for a week then every 2 days for additional 2 weeks.

### Cell lines

MDA-MB-231 and MCF7 cells were ordered through ATCC. MDA-MB-231 cells and subsequent F1 and F2 cell subpopulations were plated in medium composed of 1:1 DMEM complete media and conditional reprogramming (CR) media [13]. The CR media consists of 3:1 (v/v) complete DMEM:F12 nutrient mix supplemented with insulin (final concentration 2.5 μg/mL), gentamicin (final concentration 10 mg/mL), cholera toxin (final concentration 0.05 nM), EGF (final concentration 5 ng/mL), hydrocortisone (final concentration 200 ng/mL), adenine (final concentration 25 μg/mL), 7% fetal bovine serum and ROCK inhibitor Y-27632 (final concentration 10 μM). All cultures were maintained at 37 °C in 5% CO_2_ in a humidified chamber. Cells were split at 1-to-6 ratios every four to 5 days using Accutase cell detachment reagent (Gibco). Cell line status were confirmed via short tandem repeat analysis performed by Genetica DNA Laboratories (Burlington, North Carolina, USA).

### shRNA-mediated knockdown

shRNAs targeting the genes of interest were purchased from Sigma-Aldrich, including shRNAs targeting S100A11 (TRCN0000289926), SERPINE1 (TRCN0000370107), SRGN (TRCN0000007987), DDIT4 (TRCN0000062421), CTSD (TRCN0000003660), and MT1X (TRCN0000155121). shRNAs targeting SNRPA1 were gifted by Hani Goodarzi from the University of California, San Francisco. shRNA constructs were packaged in HEK 293 T cells using FuGENE 6 Transfection Reagent (Promega E2691) according to manufacturer’s protocol. MDA-MB-231 F2 cells were then infected with shRNA packaged within lentiviruses in Opti-MEM (Invitrogen 51,985,034) supplemented with 8 μg/mL polybrene (Millipore TR-1003-G). Once infected, cells were selected using increasing concentrations of puromycin up to 5 μg/mL as needed. Once selected, cells were kept in culture medium supplemented with basal level puromycin (0.5 μg/mL).

### 3-dimensional invasion assay

Cells were dissociated into single cells using mechanical and enzymatic dissociation via Accutase (Innovative Cell Technologies #AT 104). Once dissociated, 1000 cells/well were pipetted within a 96-well U-bottom ultra-low attachment plate (Thermo Fisher #174925) followed by a quick centrifugation step at 1200 rpm for 4 minutes. Cells were supplemented with 250 uL of medium and allowed to cluster undisturbed for 4 days. After 4 days, individual clusters were collected and centrifuged at 1200 rpm for 4 minutes at 4 °C. Once spun down, the supernatant was aspirated, and cell clusters were gently resuspended in neutralized rat tail Collagen I/BM mix (2.4 mg/mL Collagen I (Corning #354236) and 2 mg/mL Cultrex (BioTechne #3533–005-02), plated on 20 uL of a base layer of Collagen I/Cultrex, overlaied with serum free media, and allowed to invade for 24 hours. After 24 hours, embedded clusters were fixed for 1 hour at room temperature using 4% formaldehyde, followed by an additional 1 hour incubation step in 0.5% Triton X-100 diluted in PBS (v/v). Embedded clusters were stained for Phalloidin (Invitrogen, #A22283) and cell nuclei using Hoechst 33342 (Invitrogen #H3570) for 1 hour at room temperature. Immunofluorescence images were captured using a ZEISS LSM800 Laser Scanning Confocal. Finally, ImageJ analysis was used to quantify cellular invasion.

### RNA extraction and qRT-PCR

Total RNA of all cell lines was isolated using Monarch Total RNA miniprep kits (#T2010S, New England Biolabs) according to manufacturer’s protocol. RNA was extracted and measured via Nanodrop-1000. RNA was subsequently sent for either library preparation and RNA-sequencing to Genewiz or cDNA preparation via a two-step process beginning with Lunascript RT Supermix Kit (#E3010, New England Biolabs). qRT-PCR was performed on cDNA from samples for genes of interest using the Luna Universal qPCR Master Mix (#M3003, New England Biolabs) according to manufacturer’s specifications. qPCR was performed on a BioRad CFX96 Touch Real-Time detection system with 60 seconds at 95 °C followed by 40 cycles of 15 seconds at 95 °C and 30 seconds with plate read followed by a melting curve analysis. GAPDH was measured as a reference gene. Primers used are provided in Supplementary Table 2. The relative mRNA expression level was normalized to reference genes and determined using the 2^-ΔΔCT^ method.

### RNA-sequencing analysis

Library preparation and RNA-sequencing from isolated RNA samples was conducted by Genewiz, Massachusetts, USA using an Illumina sequencing platform. Only those RNA-samples that yielded a RIN score > 7.0 and sufficient RNA quantity were prepped and sequenced. Experimental design was made following consultation with Genewiz. Read files were trimmed using Trimmomatic [95] and aligned to the human genome (GRCh38.p13) using the STAR aligner [96]. Aligned reads were quantified using featureCounts [97] and differential expression analysis was performed in R using DESeq2 [98]. Normalized feature counts were used for Gene Set Enrichment Analysis (GSEA, Broad institute). Matlab R2021a was used to generate heatmaps and clustergrams for figures. In heatmaps, colors were scaled by row according to normalized feature counts. All sequencing files analyzed in this study can be found in GEO accession GSE153161.

### Differential RNA-splicing

Reads were aligned using STAR (v2.7.1a) to human genome (hg38) using iGenomes GTF annotations. MISO (v0.5.4) was then used to compare alternative splicing of skipped exons (paired end mode with fragment lengths of 219.4 (53.7) and 210.3 (52.0), for parental and derived lines respectively). Rstudio packages ggbio and ggplot2 were used to map differential splicing events and various other plots presented here.

### Protein-protein interaction and gene-disease network analysis

Network analysis for protein-protein interactions and gene-disease associations were performed using NetworkAnalyst and accessed at https://www.networkanalyst.ca [99]. Briefly, gene lists were inputted into NetworkAnalyst. Network analysis was performed investigating breast mammary tissue specific protein-protein interactions with a stringent 30.0 filter and gene-disease associations. Network visualization was performed using a Force-atlas layout and customized using the built-in network visualization toolset.

### Kaplan-Meier survival curves and clinical data

Clinical and mRNA expression data from the METABRIC study were downloaded from cbioportal.org. Expression data, clinical vital status, and clinical overall survival in months were extracted using Matlab R2019b. X-tile software was provided online by the Rimm laboratory and accessed via https://medicine.yale.edu/lab/24or/research/software/. To assess a suitable cutpoint for high/low expression data for each gene we used 700/1904 patient samples selected in un-biased fashion and used it as a “discovery cohort”. Cutpoints for each gene were determined using a dead of disease censor and 20-year cutoff for overall survival in X-tile [72]. Kaplan-Meier analysis and plots were subsequently performed in Matlab R2019b using all 1904 patient samples and the respective cutpoint for each gene.

#### Funding and acknowledgements

This study was funded in part by a grant from the ACCRF and support from the Center for Cell Reprogramming at Georgetown University to SA. In addition, it was partially supported by an appointment to the Research Participation Program at CBER, US Food and Drug Administration, administered by the Oak Ridge Institute for Science and Education through an interagency agreement between the US Department of Energy and FDA (JRM). This research was also supported by the Animal Models Zebrafish Shared Resource of the Georgetown Lombardi Comprehensive Cancer Center (P30-CA051008) as well as partial funding from NIH R01CA218670 to GP.

## Supplementary Information

**Additional file 1.**

**Additional file 2.**

## Data Availability

RNA sequencing data in the current study have been deposited in the Gene Expression Omnibus (GEO) under accession number GSE153161.
