# Supplementary material for: Identifying drivers of breast cancer metastasis in progressively invasive subpopulations of zebrafish-xenografted MDA-MB-231
Source: Mol Biomed. 2022 May 26;3:16. doi: 10.1186/s43556-022-00080-5 (PMC9133282; doi:10.1186/s43556-022-00080-5)

**Supplemental Information**

**Supplementary Table 1. STR analysis for MDA-MB-231 cell lines**

|  | TH01 | D5S818 | D13S317 | D7S820 | D16S539 | CSF1PO | vWA | TPOX | AMEL |
| --- | --- | --- | --- | --- | --- | --- | --- | --- | --- |
| From ATCC: MDA-MB-231 HTB-26 | 7, 9.3 | 12 | 13 | 8, 9 | 12 | 12,13 | 15,  18 | 8, 9 | X |
| MDA-MB-231 Parental | 7, 9.3 | 12 | 13 | 8, 9 | 12 | 12, 13 | 15, 18 | 8, 9 | X |
| MDA-MB-231 F1 (p5) | 7, 9.3 | 12 | 13 | 8 | 12 | 12, 13 | 15, 18 | 8, 9 | X |
| MDA-MB-231-F2 (p4) | 7, 9.3 | 12 | 13 | 8 | 12 | 12, 13 | 15, 18 | 8, 9 | X |

**Supplementary Table 2. Primers used in study**

| **Target** | **Forward Primer (5’ – 3’)** | **Reverse Primer (5’ – 3’)** |
| --- | --- | --- |
| **Cytokeratin 7** | CAACATCAAGAACCAGCGTG | CACAGAGATATTCACGGCTCC |
| **Cytokeratin 8** | CATAGACAAGGTACGGTTCC | CTTCATCCACATCCTTCTTG |
| **Cytokeratin 20** | AACTGAGGTTCAACTAACGGAG | CTCTTCCAGGGTGCTTAACTG |
| **EpCAM** | GTGAGAACCTACTGGATCATC | TCAGCTATGTCCACATCATTC |
| **Vimentin** | CCTGGATTTCCTCTTCGTGG | TCCGGGAGAAATTGCAGGAG |
| **SLUG** | TGCGATGCCCAGTCTAGAAA | TTCTCCCCCGTGTGAGTTCT |
| **SNAI1** | ACCCCAATCGGAAGCCTAAC | AGCCTTTCCCACTGTCCTCA |
| **ZEB1** | ACACCTTTGCATACAGAACCC | TGGTGATGCTGAAAGAGACG |
| **L1CAM** | TATGGCCTTGTCTGGGATCT | CCGGAACATCCTCTCCTTAAAC |

**Supplementary Table 3: GSEA Enrichment using Common Parental control**

| Pathway | Total | Expected | Hits | P.Value | FDR |
| --- | --- | --- | --- | --- | --- |
| TNF signaling pathway | 110 | 4.32 | 22 | 1.85E-10 | 5.90E-08 |
| Transcriptional misregulation in cancer | 186 | 7.31 | 25 | 5.75E-08 | 6.09E-06 |
| Pathways in cancer | 530 | 20.8 | 46 | 2.16E-07 | 1.49E-05 |
| NF-kappa B signaling pathway | 100 | 3.93 | 17 | 2.80E-07 | 1.49E-05 |
| MAPK signaling pathway | 295 | 11.6 | 31 | 4.50E-07 | 2.04E-05 |
| IL-17 signaling pathway | 93 | 3.65 | 16 | 5.34E-07 | 2.12E-05 |
| Cellular senescence | 160 | 6.29 | 19 | 1.49E-05 | 0.000431 |
| Cell cycle | 124 | 4.87 | 16 | 2.53E-05 | 0.000618 |
| Proteoglycans in cancer | 201 | 7.9 | 21 | 3.80E-05 | 0.000864 |
| ErbB signaling pathway | 85 | 3.34 | 12 | 0.000109 | 0.00217 |
| HIF-1 signaling pathway | 100 | 3.93 | 13 | 0.000135 | 0.00238 |
| MicroRNAs in cancer | 299 | 11.7 | 25 | 0.000277 | 0.0044 |
| Ras signaling pathway | 232 | 9.12 | 21 | 0.000296 | 0.00449 |
| Renal cell carcinoma | 69 | 2.71 | 10 | 0.000328 | 0.0046 |
| Breast cancer | 147 | 5.78 | 15 | 0.000631 | 0.00789 |
| Neurotrophin signaling pathway | 119 | 4.68 | 13 | 0.000758 | 0.0086 |
| mRNA surveillance pathway | 91 | 3.58 | 11 | 0.000823 | 0.00902 |
| Endocrine resistance | 98 | 3.85 | 11 | 0.00153 | 0.0157 |
| Rap1 signaling pathway | 206 | 8.09 | 17 | 0.00299 | 0.0265 |
| Signaling pathways regulating pluripotency of stem cells | 139 | 5.46 | 13 | 0.00313 | 0.0269 |
| Adipocytokine signaling pathway | 69 | 2.71 | 8 | 0.00538 | 0.0392 |
| PI3K-Akt signaling pathway | 354 | 13.9 | 24 | 0.00622 | 0.043 |
| mTOR signaling pathway | 153 | 6.01 | 13 | 0.00706 | 0.0468 |
| Endometrial cancer | 58 | 2.28 | 7 | 0.00727 | 0.0472 |
| Toll-like receptor signaling pathway | 104 | 4.09 | 10 | 0.00752 | 0.0478 |
| Estrogen signaling pathway | 138 | 5.42 | 12 | 0.00793 | 0.0495 |
| DNA replication | 36 | 1.41 | 5 | 0.0126 | 0.0726 |
| FoxO signaling pathway | 132 | 5.19 | 11 | 0.0146 | 0.0798 |
| Chemokine signaling pathway | 190 | 7.47 | 14 | 0.0173 | 0.0934 |
| Hippo signaling pathway | 154 | 6.05 | 12 | 0.0179 | 0.095 |
| Ubiquitin mediated proteolysis | 137 | 5.38 | 11 | 0.0188 | 0.0962 |
| Insulin signaling pathway | 137 | 5.38 | 11 | 0.0188 | 0.0962 |
| RIG-I-like receptor signaling pathway | 70 | 2.75 | 7 | 0.0195 | 0.0983 |
| VEGF signaling pathway | 59 | 2.32 | 6 | 0.0275 | 0.123 |
| GnRH signaling pathway | 93 | 3.65 | 8 | 0.0293 | 0.126 |
| EGFR tyrosine kinase inhibitor resistance | 79 | 3.1 | 7 | 0.035 | 0.145 |
| Notch signaling pathway | 48 | 1.89 | 5 | 0.0391 | 0.155 |
| Regulation of actin cytoskeleton | 214 | 8.41 | 14 | 0.0421 | 0.162 |
| Sphingolipid signaling pathway | 119 | 4.68 | 9 | 0.0442 | 0.167 |

**Supplementary Table 4: Top 20 genes with enriched RNA-splicing in F1/F2 populations**

| Gene Symbol | Splice Variant | Difference | Bayes Factor |
| --- | --- | --- | --- |
| ATG5 | chr6:106279661:106279823:-@chr6:106248150:106248240:-@chr6:106201972:106202089:- | 0.79 | 1.00E+12 |
| RBBP9 | chr20:18493958:18494063:-@chr20:18490395:18490480:-@chr20:18486540:18489990:- | 0.76 | 1.00E+12 |
| FRMD8 | chr11:65389361:65389528:+@chr11:65393573:65393674:+@chr11:65394041:65394099:+ | 0.66 | 496.72 |
| RFFL | chr17:35026374:35026561:-@chr17:35021371:35021781:-@chr17:35017523:35017606:- | 0.65 | 8295532 |
| PTK2 | chr8:140700891:140701022:-@chr8:140686632:140686694:-@chr8:140675460:140675499:- | 0.65 | 1261.36 |
| SLFNL1-AS1 | chr1:41037362:41037541:-@chr1:41033983:41034048:-@chr1:41028584:41028726:- | 0.63 | 118.81 |
| TMEM39B | chr1:32075603:32075822:+@chr1:32077164:32077318:+@chr1:32091675:32092011:+ | 0.61 | 20.04 |
| SPAG9 | chr17:50977108:50977221:-@chr17:50975863:50975901:-@chr17:50974771:50974947:- | 0.61 | 35423877 |
| TMEM182 | chr2:102764329:102764427:+@chr2:102797863:102798000:+@chr2:102814748:102817679:+ | 0.61 | 61.54 |
| PEX11A | chr15:89690577:89690782:-@chr15:89686431:89686546:-@chr15:89681531:89683948:- | 0.61 | 26725.48 |
| PNISR | chr6:99404603:99404702:-@chr6:99403829:99403882:-@chr6:99402540:99402710:- | 0.6 | 13.66 |
| TRIM39-RPP21 | chr6:30345298:30345398:+@chr6:30345491:30345929:+@chr6:30346432:30346557:+ | 0.59 | 535.64 |
| STAU2 | chr8:73738284:73738349:-@chr8:73709032:73709162:-@chr8:73688654:73688813:- | 0.59 | 85.84 |
| TRPC1 | chr3:142724074:142724731:+@chr3:142736379:142736533:+@chr3:142748258:142748460:+ | 0.59 | 9.32 |
| TM2D1 | chr1:61694697:61694770:-@chr1:61686792:61686921:-@chr1:61683417:61683546:- | 0.58 | 8.42 |
| METTL27 | chr7:73841070:73841198:-@chr7:73840414:73840549:-@chr7:73840031:73840120:- | 0.58 | 9.39 |
| LPAR1 | chr9:111037841:111038085:-@chr9:110973481:110973558:-@chr9:110972073:110972220:- | 0.58 | 7.29 |
| COPA | chr1:160299102:160299264:-@chr1:160298845:160298931:-@chr1:160297556:160297745:- | 0.57 | 7.06 |
| ABCB9 | chr12:122944391:122944519:-@chr12:122940807:122940995:-@chr12:122940111:122940284:- | 0.57 | 17.68 |
| MTRR | chr5:7870770:7870923:+@chr5:7873373:7873526:+@chr5:7875258:7875375:+ | 0.56 | 1.25E+10 |

**Supplementary Video 1: Mixed MDA-MB-231 parental + F2 cell clusters demonstrating trailblazer phenotype for F2 cells.** RFP-labeled F2 and GFP-labeled parental cells were co-clustered and embedded in 3D ECM showing F2 cells invade the ECM progressively with time, but not parental cells. Time recorded is from 0-24 h.

**Supplementary Fig. 1:** Kaplan-meier survival curves evaluating associations between (a) CTSD, (b) DDIT4, (c) MT1X, (d) S100A11, (e) SERPINE1, (f) SNRPA1, and (g) SRGN from the METABRIC dataset.


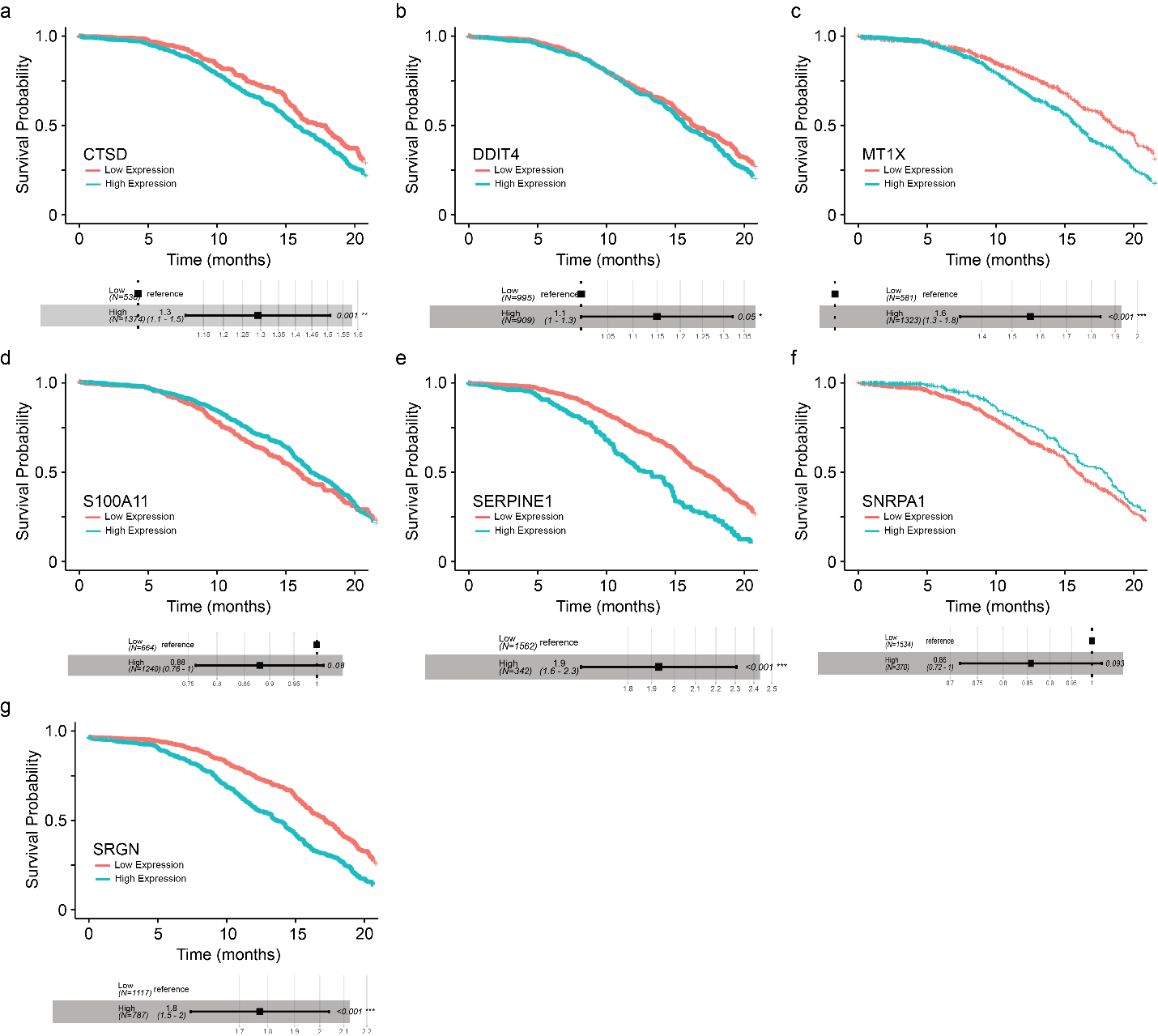

Supplement: Supplementary file 2 — Additional file 2. [file 43556_2022_80_MOESM2_ESM.docx]
